# Machine Learning-Driven Prediction of Reactive Oxygen Species Dynamics for Assessing Nanomaterials’ Cytotoxicity

**DOI:** 10.3390/biomimetics10110718

**Published:** 2025-10-24

**Authors:** Zuowei Ji, Ziyu Yin

**Affiliations:** 1 School of Humanities and Social Sciences, The Chinese University of Hong Kong, Shenzhen 518172, China; 2 Shenzhen Key Laboratory of Nano-Biosensing Technology, Marshall Laboratory of Biomedical Engineering, International Health Science Innovation Center, School of Biomedical Engineering, Shenzhen University Medical School, Shenzhen University, Shenzhen 518060, China; z_yin@szu.edu.cn

**Keywords:** nanomaterials, reactive oxygen species, oxidative stress, machine learning

## Abstract

Nanomaterials (NMs) possess unique physicochemical features that set them apart from bulk counterparts. Their adjustable properties provide remarkable flexibility, giving rise to a wide array of variants. However, these attributes can also trigger complex biological interactions, particularly the generation of reactive oxygen species (ROS), which are central to nanomaterial-induced cytotoxicity. The ambivalent nature of ROS, essential for physiological signaling yet harmful when dysregulated, can lead to substantial health consequences. The scarcity of reliable toxicity and safety data, together with the inadequacies of conventional testing methods, highlights the urgent need for more effective strategies to assess nanomaterial-related hazards and risks. Given the intricate interplay between NMs and biological systems, computational approaches, particularly machine learning (ML), have emerged as powerful tools to model ROS dynamics, predict cytotoxic outcomes, and optimize nanomaterial design. This review highlights recent advances in applying ML to predict both the generation and neutralization of ROS by diverse NMs and to identify the critical determinants underlying ROS-mediated toxicity. These insights provide new opportunities for predictive nanotoxicology and the development of safer, application-tailored NMs.

## 1. Introduction

Nanomaterials (NMs), typically characterized by at least one dimension within the 1–100 nm range, exhibit distinctive physicochemical behaviors that differ markedly from their bulk counterparts, largely attributable to their nanoscale size. Features such as an exceptionally high surface area-to-volume ratio, quantum confinement effects, and inherent biocompatibility render them highly advantageous for applications spanning electronics, biomedicine, energy conversion, environmental remediation, etc. [1,2,3,4,5] Moreover, the physicochemical characteristics of NMs can be deliberately engineered to meet targeted functional demands, yielding an almost limitless diversity of NM variants. To harness the full potential of these tailored NMs, the establishment of effective hazard and risk assessment frameworks is imperative.

**Figure 1:**
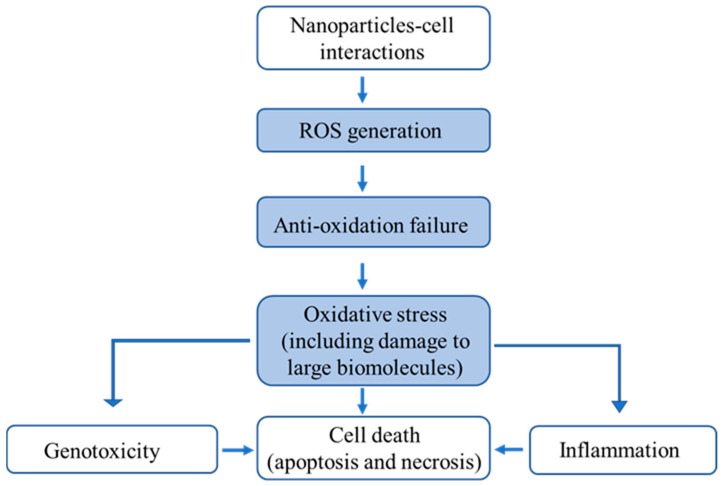
The cytotoxicity resulting from excessive ROS production by nanoparticles (NPs) [27].

Regarding nanotoxicity, the induction of reactive oxygen species (ROS) and the resultant oxidative stress are widely regarded as central mechanisms underlying the adverse effects of NMs [6,7,8,9,10,11,12,13,14]. ROS encompass a variety of oxygen-derived reactive species, including hydroxyl radicals (·OH), superoxide anions (O_2_^·−^), and hydrogen peroxide (H_2_O_2_), which serve a dual role in biological systems. While they function as critical signaling mediators in processes such as proliferation, differentiation, and immune defense, excessive ROS accumulation can provoke oxidative stress, leading to DNA damage, disrupted cellular communication, cytotoxicity, and even carcinogenesis [15,16,17,18]. Figure 1 schematically illustrates how exposure to NMs induces oxidative stress through excessive ROS generation, ultimately leading to cellular damage and cytotoxicity. NMs interact with cellular membranes and organelles, triggering excessive ROS production via surface redox reactions and mitochondrial dysfunction. When ROS overwhelms antioxidant defenses, oxidative stress damages lipids, proteins, and DNA, leading to inflammation, genotoxicity, apoptosis, or necrosis. Maintaining a delicate balance of ROS within cells is therefore essential for homeostasis and overall health. Experimental studies have shown that NMs can either promote or suppress ROS generation through distinct intracellular mechanisms [19,20,21,22,23,24,25]; for example, titanium dioxide (TiO_2_) nanoparticles (NPs) have been associated with oxidative stress in BEAS-2B cells, potentially linked to lipid metabolism alterations, with several metabolites—including 7-dehydrocholesterol, eicosapentaenoic acid, montecristin, azaspiracid, and cincassiol B—identified as being strongly correlated with ROS dysregulation [26].

However, the physicochemical parameters that govern ROS generation or scavenging differ significantly across NMs types [28]. The immense diversity of nanomaterial variants presents a significant challenge that conventional experimental methods, constrained by high costs, slow timelines, and limited predictive scope, cannot efficiently address. Computational tools mitigate these inefficiencies by enabling rapid in silico screening, deciphering complex relationships in high-dimensional data, and providing predictive insights into biological interactions, such as ROS dynamics across diverse scenarios.

**Figure 2:**
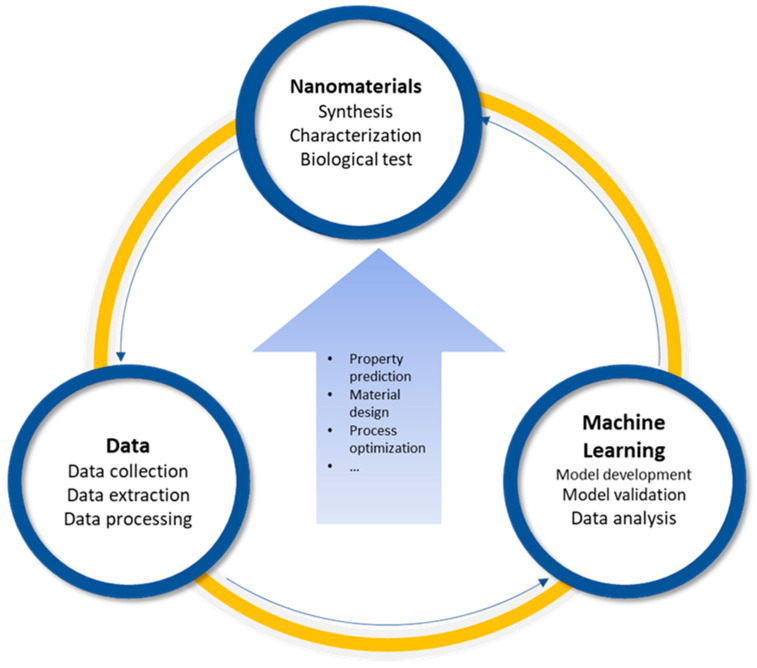
The significance of machine learning to advance nanotechnology.

For instance, titanium dioxide (TiO_2_) NMs have been extensively studied through density functional theory (DFT), which enables the accurate prediction of crystal structures and their associated photocatalytic properties [29]. To further address these challenges and deepen our mechanistic understanding, machine learning (ML), a core component of artificial intelligence (AI), has been increasingly applied to diverse tasks in the NMs field, including property prediction, material design, and process optimization [30,31,32,33,34,35,36,37,38,39,40,41,42,43] (Figure 2). Take the nanotoxicity as an example, researchers have constructed quantitative structure-activity relationship (QSAR) models based on datasets of 30 distinct metal oxide nanoparticles (MeONPs) to predict their inflammatory potential [44]. Complementary DFT analyses further revealed that MeONPs with lower metal electronegativity and positive f-potential were more prone to disrupt lysosomal integrity, thereby triggering inflammatory responses. Building on these advances, this review provides a comprehensive overview of how ML is being integrated to investigate and forecast the ROS-related behaviors of primary types of NMs—including metal oxide, metal-based, and carbon-based NMs [45]—with a particular emphasis on their capacities to induce or scavenge ROS.

This manuscript that details the application of ML algorithms in the realm of NMs research reveals a highly promising convergence with biomimetics, particularly in the development and design of bioinspired, biomedical materials at the nanoscale. The adeptness of ML in identifying complex patterns and delivering precise predictions from vast datasets dovetails perfectly with the objectives of biomimetics, which focus on the application of biological principles for pioneering new nanotechnologies. By employing ML to decipher and predict the behaviors and characteristics of NMs, researchers can significantly expedite the innovation cycle, learning crucial structure-property relationships that lead to the creation of more effective and inherently safer NMs.

Our focus on “predictive nanotoxicology” and the creation of “safe-by-design NMs” plays a pivotal role in the biomedical applications of these novel materials. This approach ensures the development of technologies that are not only effective, but that are also inherently biocompatible and safe, aligning with the journal’s overarching aim of fostering “sustainable innovation” in the biomedical sector. These initiatives underscore the critical integration of ML-driven strategies with biomimetic principles, setting a course for transformative advances that extend beyond traditional technological approaches and contribute significantly to the advancement of biomedicine and materials science.

## 2. Literature Survey

A comprehensive literature survey was conducted using Google Scholar as the primary database, applying the search terms ‘”nanomaterials” AND “oxidative stress” OR “oxidative damage” OR “ROS” AND “machine learning”’ to retrieve relevant articles published within the timeframe from 2015 through July 2025. The search strategy was inclusive, with no restrictions imposed on article type, category, or publishing source.

## 3. Machine Learning Aids in the Investigation of ROS Induction in Primary Types of NMs

### 3.1. Metal Oxide (MeOx) NMs

Metal oxide NMs are extensively utilized due to their distinctive optoelectronic properties and mechanical robustness. Their widespread application has motivated the development of cheminformatics-based models to evaluate potential toxicological risks [46,47].

One such approach employed a nano-QSAR framework with quasi-SMILES descriptors to identify key physicochemical determinants of toxicity across different classes of MeOx NMs (MeO, MeO_2_, MeO_3_, and Me_2_O_3_) [48]. The findings highlighted oxidative stress as the dominant mechanism of toxicity in *C. elegans*, with those possessing higher absolute zeta potential or elevated metal cation charge inducing more intense oxidative damage.

In a study on TiO_2_ NPs, 36 ML models were constructed using six algorithms and multiple nanodescriptors, including NP size, exposure concentration, exposure duration, and type of assay organisms/tissues/organs to explore the toxicological mechanisms of nTiO_2_ in aquatic environments, with a focus on bivalves [49]. Each ML model was paired with a specific biological assay, and among the top-performing models, four algorithms—random forest (RF), artificial neural network (ANN), k-nearest neighbor (KNN), and eXtreme Gradient Boosting (XGB) were most prominent. Notably, RF demonstrated the highest frequency of selection and superior predictive performance compared to the others. The study further revealed that elevated doses of nTiO_2_ combined with short exposure durations were especially harmful, resulting in pronounced oxidative stress.

Another study examined the cytotoxicity of nano-TiO_2_ in combination with eight heavy metals on human kidney (HK-2) cells [50]. To predict cell viability and elucidate mechanisms of ROS-mediated cytotoxicity, ML-based QSAR models were constructed using Partial Least Squares (PLS), RF, Adaptive Boosting (AdaBoost), and a hybrid approach integrating K-means clustering with RF. Among these, the RF-K-means model demonstrated the highest predictive accuracy, establishing it as a reliable tool for modeling the cytotoxicity of nano-TiO_2_-heavy metal mixtures. Mechanistic analysis revealed that co-exposure markedly increased hydroxyl radicals (·OH) production, thereby inducing oxidative stress and apoptosis. Furthermore, quantum mechanical descriptors such as lowest orbital energy, absolute hardness, and adsorption energy were identified as key parameters for reliably predicting ROS-mediated cytotoxicity.

Le et al. applied two modeling approaches including linear regression and Bayesian regularized neural networks to analyze a dataset comprising 45 ZnO NPs [51]. The nonlinear ML models outperformed the linear ones, achieving an R^2^ value of 0.67 in predicting oxidative stress. Critical descriptors driving oxidative stress included the dopant materials’ ionization potential (IP), redox potential (RP), along with the conduction band energy (Ec). Moreover, these nonlinear models exhibited robust predictive capabilities regarding cell viability and membrane integrity.

A study examining the impact of superparamagnetic iron oxide nanoparticles (SPION) surface properties, particularly coatings, on toxicity and biocompatibility developed a partial least squares regression (PLSR) model as an effective analytical framework [52]. This model linked the physicochemical characteristics of SPION coatings with their toxicological responses using predefined descriptors. Its performance was validated by a high coefficient of determination (R^2^ = 0.9515) and a low mean squared error of cross-validation (MSECV = 0.6270). Variable Importance in Projection (VIP) scores were further calculated to aid the selection of relevant predictors under conditions of multicollinearity. Notably, the number of non-hydrogen bonds in a molecule was associated with toxicity, reflecting the role of molecular complexity and reactivity in promoting ROS production. Similarly, higher surface charge was found to enhance polarization and intensify interactions with the macromolecular environment, frequently triggering ROS generation and thus elevated cytotoxicity.

Another study utilized ML models to quantitatively predict the cytotoxicity of metal oxide nanoparticles (MeONPs) in macrophages [39]. Ten algorithms were evaluated, encompassing regression and ensemble methods, including polynomial regression, multivariate polynomial regression, group multivariate polynomial regression, support vector machine (SVM) regression, RF regression, KNN regression, group KNN regression, decision tree (DT) regression, Bayesian regression, and a hybrid model integrating neural networks with RF. Feature importance was examined using SHAP (Shapley Additive Explanations) analysis, which highlighted five critical features of macrophage toxicity: exposure concentration, degree of dissociation in a lysosome-simulating PSF solution, primary particle size, ζ-potential in water, and electronegativity. Collectively, the results emphasized that exposure dose, ion release potential, particle size, surface charge, and electronegativity are pivotal physicochemical properties governing ROS-related cytotoxicity of MeONPs. Among the evaluated algorithms, group KNN regression demonstrated the highest reliability, offering robust generalization capability and enhanced mechanistic interpretability. These insights significantly advance the field of computational toxicology by elucidating the intricate relationships between nanoparticle physicochemical properties, ROS generation, and subsequent biological impacts.

### 3.2. Metal-Based NMs

Recent research has increasingly focused on metallic NMs, which are highly valued for their distinctive properties that support applications in catalysis [53], sensing [54], and biomedical diagnosis and therapy [55].

Yan et al. proposed an innovative computational workflow for virtual of NP profiling that included (1) the construction of a structurally diverse virtual library of gold nanoparticles (GNPs) and (2) the development of universal nanodescriptors applicable to nanomodeling and virtual screening, thereby facilitating the rational design of NMs [56]. Utilizing these nanodescriptors, kNN and RF algorithms were applied to build two quantitative nanostructure-activity relationship (QNAR) models for predicting ROS induction in HEK293 cells. The CNOM descriptor, which our feature importance analysis identified as the most effective predictor of ROS induction, was designed to quantify the local electronic heterogeneity of the nanoparticle surface. It is calculated using a Delaunay tessellation framework that decomposes the nanoparticle surface into tetrahedral clusters of neighboring carbon (C), nitrogen (N), oxygen (O), and gold (Au) atoms. The descriptor’s value represents the frequency-weighted sum of Pauling electronegativities within these tetrahedra, effectively capturing how different atomic arrangements create reactive sites for electron transfer. The analysis revealed that CNOM was the most influential feature, particularly for nanoparticles with acyl- or amine-containing ligands, confirming its utility in representing the specific surface chemistry that drives oxidative potential. Beyond ROS prediction, these QNAR models were further extended to evaluate additional nano-bioactivities and physicochemical properties.

Wang et al. constructed a QNAR model utilizing 11 descriptors and the kNN algorithm to predict the potential of GNPs to induce oxidative stress [57]. The model achieved high predictive accuracy, with correlation coefficients (R^2^) of 0.967 and a mean absolute error (MAE) of 0.14. Feature analysis identified four descriptors as the most influential in predicting oxidative stress, including the number of surface ligands, propensity for nonbonded hydrophobic interactions, interactions potential with water molecules, and electrostatic positivity.

A comprehensive meta-analysis employed advanced ML models, specifically DT and RF, to identify the principal determinants of cytotoxicity in phytosynthesized silver nanoparticles (AgNPs) [58]. The models incorporated an extensive range of descriptors, encompassing nanoparticles’ physicochemical properties, biological characteristics, exposure conditions, and synthesis-related factors. The findings revealed that the RF model provided superior predictive performance, particularly for cytotoxicity driven by ROS-related mechanisms. Key features contributing to its accuracy included exposure regime, cell type, plant family, particle size, and surface charge.

MXenes, a class of two-dimensional (2D) transition metal carbides and nitrides, represent a rapidly advancing area of research in 2D materials. Marchwiany et al. developed ML models to predict the cytotoxicity of MXenes [59]. The analysis identified transition metal oxides and lithium atoms on the MXene surface as primary determinants of ROS production and oxidative stress, with additional factors such as surface modifications, delaminating agents, lateral size, and surface chlorine serving as secondary contributors. Among the algorithms evaluated, SVM with a radical basis function kernel and RF yielded the highest predictive performance.

### 3.3. Carbon Nanomaterials (CNMs)

Carbon nanomaterials, including carbon dots, fullerenes, carbon nanotubes, graphene, and graphene oxide, derive their distinctive structural features from sp^2^/sp^3^-hybridized carbon atoms [60,61]. Their unique physicochemical properties have attracted considerable research interest, particularly regarding their intricate interactions with ROS [62,63].

An ANN model based on a multilayer perceptron (MLP) algorithm was developed to predict metabolic stress responses in hairy root cultures of Evolvulus alsinoides, a medicinally valuable shrub, following exposure to carbon dots (CDs) [64]. The study measured H_2_O_2_ levels as well as the activity of antioxidant enzymes such as superoxide dismutase and peroxidase enzymes, both of which play pivotal roles in ROS-related biochemical pathways essential for mitigating oxidative stress in the body. The ANN model demonstrated excellent predictive performance, achieving a mean squared error (MSE) of 1.99 × 10^−3^ and a coefficient of determination (R^2^) of 0.99939 across both training and testing datasets.

To identify key determinants of ROS generation, Wang et al. built a virtual library of graphene structures using nanostructure annotation methods [65]. PLSR models were then developed and validated through leave-one-out cross-validation (LOOCV), yielding an R^2^ of 0.760 for ROS generation and a root mean square error (RMSE) of 0.164 for the test set. Among the evaluated nanodescriptors, the surfactant descriptor PF108 was identified as the most influential factor driving oxidative stress, likely by enhancing the dispersibility and stabilization of two-dimensional nanomaterials. Beyond ROS prediction, these models also exhibited strong accuracy in forecasting additional nanotoxicity endpoints, including apoptosis, cell viability, and LDH release. This structural annotation approach underscores the potential of ML to predict the toxicity of NMs with complex nanostructures.

A recent study developed multiple nano-QSAR models to predict the oxidative stress potential of various CNMs in algal cells, both individually and in binary combinations [66]. These models integrated ML algorithms with experimental toxicity data, employing both classification and regression approaches. For classification, the objective was to ascertain whether CNMs could induce ROS, using five distinct ML algorithms: C4.5 DT, support vector machine (SVR), ANN, naïve Bayes, and KNN. Enhanced models integrating zeta potential (fP), hydrodynamic diameter (DH), and specific surface area (SSA) with logistic regression, RF, and AdaBoost improved predictive performance, with all classification metrics (accuracy, recall, precision, F1-score) exceeding 0.600 Regression models were developed to quantify ROS levels, incorporating fP, DH, and SSA with DT regression, RF regression, gradient boosting (GB), and Adaboost. These models achieved strong goodness-of-fit, with R^2^ values ranging from 0.850 to 0.999 on training set, signifying their capacity to explain variability in ROS generation. Experimentally, most CNMs except graphene oxide (GO), produced higher ROS levels than the control, with multi-walled carbon nanotubes (MWCNTs) exhibiting the strongest ROS induction. For binary mixtures, certain combinations, notably fullerene with graphene nanosheets and graphene nanosheets with single-walled carbon nanotubes (SWCNTs), produced synergistic effects, generating ROS levels surpassing those of their individual components, while other mixtures showed variable or attenuated ROS responses.

### 3.4. Mixed Types of NMs

Researchers have applied DT models to categorize NMs within the DF4nanoGrouping framework, which organizes NMs according to their physicochemical characteristics and biological interactions [67]. A wide set of descriptors (285 in total) was evaluated, and the model based solely on the ‘Size’ descriptor achieved 100% balanced accuracy in predicting the intrinsic oxidative potential of NMs. Two additional DT models, centered on protein carbonylation and short-term inhalation, also demonstrated low complexity, requiring only a limited number of descriptors while maintaining strong robustness, underscoring their utility in supporting the DF4nanoGrouping approach.

In another research, a self-organizing map (SOM) model was developed to analyze and predict NPs toxicity [68]. The SOM first evaluated toxicity similarities among metal and silica oxide NPs, categorizing them into distinct clusters. Subsequent QSAR analysis revealed hidden toxicity patterns and identified key determinants, notably oxidative stress arising from excessive ROS generated either by ions release or NP surfaces.

To investigate oxidative stress associated with DNA damage induced by TiO_2_, ZnO, silver, and silica NMs, the supervised partial least squares (PLS) method was applied [69]. The QSAR analysis indicated that coated NMs were more likely to induce oxidative DNA damage compared to their uncoated counterparts. Cheminformatics modeling further identified electron properties and overall chemical reactivity as critical descriptors of cytotoxic potential, while HOMO-LUMO energy parameters, ionization potential, and pristine particle size were highlighted as key determinants of genotoxic potential.

Another work developed an ML framework to predict the in vitro cytotoxicity of inorganic NPs, offering a significant improvement over conventional binary toxic/non-toxic classifiers [70]. Traditional models often neglect the complex dose–response relationships central to understanding ROS-mediated toxicity. Incorporating over 3000 meticulously curated data samples, the framework incorporated atom-based descriptors, detailed physicochemical features, and experimental parameters. Among 40 evaluated models, the Light Gradient Boosting Machine (LightGBM) regressor achieved the best performance, with a predictive quality (Q^2^) of 0.86 and a root mean square error (RMSE) of 12.2%. SHAP analysis further enhanced interpretability, revealing key determinants such as concentration- and time-dependent viability loss, enhanced cellular uptake of smaller particles, and the pivotal role of elemental electronegativity in driving ROS production.

To predict NP-induced cytotoxicity, researchers analyzed a dataset of 244 records from the NanoHub repository, encompassing metallic, metal oxide, polymeric, and silica NPs [71]. ML techniques, particularly the RF algorithm, demonstrated strong performance in predicting ROS-mediated cytotoxicity. The model leveraged NP physicochemical properties, exposure conditions, and cell model characteristics. Key predictors identified included cell line susceptibility, exposure dose, and tissue type, emphasizing their central role in determining ROS-related toxicity.

A recent study combined ML with a reinforced genetic algorithm to predict and screen selectively cytotoxic inorganic NPs [72]. An extensive dataset was compiled from over 100 publications, encompassing more than 3700 samples and retaining 27 critical features for analysis. The pivotal determinants of NP cytotoxicity included concentration, where higher doses escalate ROS production and cellular damage; zeta potential, with more positive charges enhancing nanoparticle-cell membrane interactions and ROS stress; hydrodynamic diameter, where smaller particles more effectively penetrated cells to elevate ROS levels; exposure time, where prolonged contact raised intracellular ROS accumulation; and the electronegativity of the central atom, with higher values associated with stronger oxidative stress and reduced cell viability. Among 41 regression models tested, XGB and LightGBM achieved the best predictive performance. The study also identified silver (Ag), copper oxide (CuO), and gold (Au) NPs as particularly potent ROS inducers in cancer cells, attributed to their rapid cellular uptake and metabolic processing.

## 4. Machine Learning Aids in the Investigation of ROS Scavenging in Primary Types of NMs

For the ROS scavenging session, given the limited number of studies, relevant reports are discussed collectively. The antioxidant capacity of NMs, defined as their ability to neutralize/scavenge free radicals, serves as a critical indicator in this context.

Mejía-Méndez et al. evaluated the antioxidant activity of (La, Sm)-doped ZnO NPs using eight distinct regression models, including LR, MLP, RF, DTs, extremely random trees (ETs), KNNs, GB, and SVM [73]. Amongst these, the GB model yielded the highest predictive performance, with a mean absolute error (MAE) of 9.027 and R^2^ = 0.86. Feature importance analysis further identified the DPPH method, NP concentration, texture coefficient (TC), and the material charge as the most influential variables.

In another study, eight ML algorithms were utilized to analyze the cytotoxicity and antioxidant activity of (Er, Yb)-doped ZnO NPs based on their physicochemical properties [74]. Among the tested models, ETs and DT achieved the highest accuracy in predicting antioxidant activity, whereas SVR and LR performed the worst, indicating that antioxidant activity follows a highly nonlinear pattern. Feature importance analysis further highlighted NP concentration, optical bandgap, and surface properties as the most influential factors governing antioxidant activity.

For neodymium (Nd)-doped ceric oxide (CeO_2_) NPs, the incorporation of Nd was found to significantly enhance antioxidant capacity [75]. To further elucidate this effect, multiple ML approaches were applied, among which the RF model achieved the highest accuracy of 96.35% with an error rate of 3.65%. This establishes RF as a practical computational tool for predicting the antioxidant activity of Nd-doped CeO_2_ NPs. Feature importance analysis revealed NP concentration as the most influential factor, followed by the methods used to access antioxidant capacity. Although both methods provide complementary insights, their differing levels of importance likely arise from variations in reaction kinetics.

In another study, a dataset derived from 62 in vitro experiments was used to develop multiple regression models predicting the antioxidant efficiency of both inorganic and organic NMs [76]. Among the evaluated models, the RF algorithm demonstrated the strongest predictive performance, achieving an R^2^ value of 0.83. Attribute importance analysis further identified NM type, core size, and dosage as the most critical factors influencing predictive outcomes.

Nanozymes, a class of NMs that mimic enzymatic activity, have garnered significant attention for their enhanced durability and cost-effectiveness compared to natural enzymes [77]. In this field, ML techniques have been increasingly employed to forecast catalytic behaviors, encompassing peroxidase-like (POD-like), oxidase-like, and catalase-like (CAT-like) functions [78,79].

One study, for instance, developed adsorption energy-based descriptors combined with ML models to efficiently predict POD- and CAT-like activities [80]. The ML framework utilized 66 structural and elemental features derived from unit-cell properties of the materials. By estimating adsorption energies of ROS intermediates, these ML models can ascertain whether a material acts as a ROS generator (pro-cytotoxic) or a ROS regulator (anti-oxidative). Among the tested algorithms, XGB regression outperformed others, including such as linear regression (LR), ridge regression (RR), SVM, KNN, gradient boosting regression (GBR), RF, and neural networks (NN). These results highlight ML as a powerful virtual screening tool for assessing the cytotoxicity and regulatory potential of nanozyme materials.

A recent study introduced a high-throughput computational screening workflow that integrates DFT calculations with ML to evaluate the POD-like activities of bimetallic alloy NPs [81]. These activities involve the catalytic dissociation of H_2_O_2_, thereby reducing ROS levels. A novel descriptor, termed “Mo-Sl” (motif and slab), was introduced to capture essential features of the alloys, including lattice constant, electronegativity, atomic number, and adsorption energy. The CatBoost algorithm was employed for optimal regression performance. The analysis revealed that alloys containing Au, Ag, Pd, and Pt generally exhibit high POD-like activity, whereas those incorporating Cu, Rh, Ir, or Ru tend to show lower activity. Furthermore, a volcano-shaped relationship was identified between O* adsorption energy and activation free energy, directly correlating with POD-like activity. Feature importance analysis within the Mo-Sl descriptor highlighted lattice constant as the most influential factor affecting O* adsorption energy.

A recent study established a machine learning-density functional theory (ML-DFT) framework to systematically screen and identify multifunctional biomaterials with strong anti-tumor and anti-inflammatory activities [82]. Using a comprehensive database, ML models were trained to predict biomaterial properties, while SHAP were applied to elucidate the contribution of individual features. Several regression algorithms were evaluated, including RF, XGBoost, AdaBoost, multilayer perceptron (MLP), and convolutional neural network (CNN). Among these, CNN demonstrated the best performance, achieving 80% accuracy in predicting CAT-like activity, which reflects the ability of materials to catalyze H_2_O_2_ decomposition into water and oxygen. Sensitivity analysis further identified the number of valence electrons in the primary metal as the most influential factor, highlighting the critical role of electronic structure in governing the enzyme-mimicking behavior of these biomaterials.

Another study employed DFT calculations to elucidate atomistic mechanisms by which inorganic NMs catalyze cellular ROS transformations, including H_2_O_2_ activation, H_2_O_2_ dismutation, O_2_ activation, and O_2_^·−^ dismutation [83]. Kinetic equations and predictive models were developed to quantitatively link NM surface structures with catalytic functions. For H_2_O_2_ activation and dismutation, hydroxyl adsorption energy (Eads, OH) and hydrogen adsorption energy (Eads, H) were identified as critical descriptors, with defined energy windows determining whether a material is active and selective for these reactions. The O_2_^·−^ dismutation model, in turn, was based on frontier molecular orbital (FMO) energy levels, which must align with the electrochemical potentials of ROS redox pairs for a material to act as an effective antioxidant. These predictive models were subsequently integrated into a ML-based computational framework, enabling high-throughput screening of candidate NMs for cancer catalytic therapy. This approach not only clarified the mechanistic principles underlying NM-mediated ROS conversation but also provided a theoretical foundation for the virtual design and selection of NMs within therapeutic potential in oncology.

## 5. Discussion

**Figure 3:**
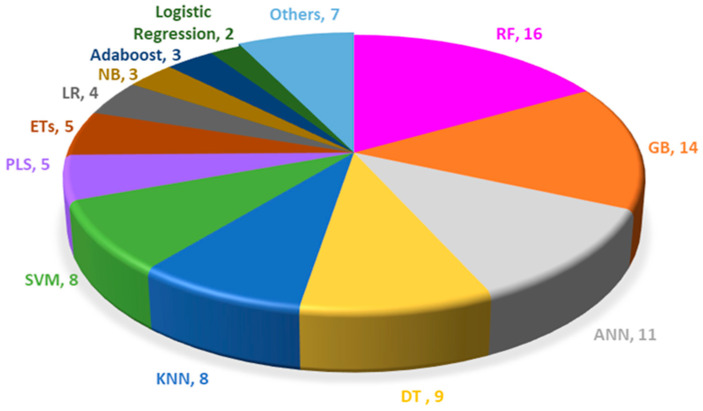
The distribution of algorithms used across the reviewed studies. ANN: artificial neural network; RF: random forest; GB: gradient boosting; KNN: k-nearest neighbor; DT: decision tree; SVM: support vector machine; PLS: partial least squares; LR: linear regression; ETs: extremely random trees; NB: naive bayes; Others: other algorithms.

Table 1 and Table 2 summarize the ML algorithms applied to evaluate the capacity of primary types of NMs to induce or scavenge ROS. In addition, Table A1 and Figure 3 illustrate the distribution of algorithms across the reviewed studies, revealing a clear preference for certain models. Notably, RF, GB, ANN, DT, KNN, and SVM are more frequently adopted than others. This trend reflects the superior ability of supervised learning models to capture complex interactions between descriptors and ROS-related outcomes compared to unsupervised learning models, which generally lack the direct ability to handle nonlinear relationships in the context of predictive modeling. Among the supervised learning models, RF and GB are most commonly used as their ensemble methods enhance robustness and predictive accuracy by aggregating multiple decision trees, thereby minimizing overfitting. Meanwhile, ANN-based models, with their deep learning capabilities, represent an advanced strategy for optimizing and predicting biological responses. Their effectiveness and reliability have been increasingly validated in recent studies [84,85]. In terms of DT, KNN, and SVM, they are relatively efficient with smaller datasets compared to more complex models such as ANNs. It is important to note that the performance of KNN heavily depends on the choice of ‘k’ and the distance metric used.

**Figure 4:**
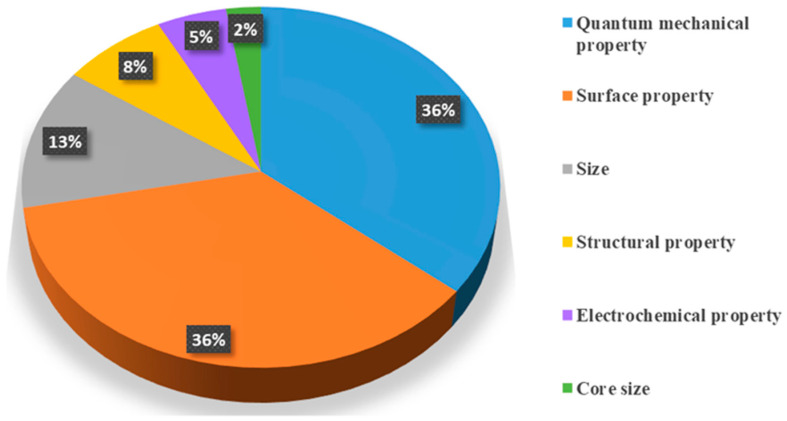
The frequency of various intrinsic factors influencing oxidative stress, as mentioned in the reviewed articles.

Furthermore, both extrinsic and intrinsic factors influencing oxidative stress have also been examined. Among extrinsic factors, exposure duration and object of study were noted, with NM dosage most frequently emphasized in the reviewed literature (Table A2). Intrinsic factors, including the structure, size, and other physicochemical characteristic of NMs, showed varying capacities to induce or scavenge ROS. To illustrate their relative importance, Figure 4 presents a pie chart summarizing the distribution of intrinsic factors identified as critical drivers of oxidative stress across the reviewed studies. Notably, quantum mechanical properties—such as ionization potential, molecular orbitals, and energy levels—and surface chemistry characteristics, including factors like the type and number of surface ligands, zeta potential, and coating type, have emerged as the most consistently reported determinants. For instance, a material’s ionization potential governs its tendency to donate electrons. Consequently, NMs with a low IP readily lose electrons, enabling them to function as antioxidants by neutralizing reactive species. Similarly, surface chemistry is critical. Surface ligands can be engineered to modify key physicochemical properties of NMs, such as hydrophilicity and zeta potential. An effective antioxidant nanomaterial, for example, typically requires a hydrophilic surface to ensure good dispersion and bioavailability, thereby maximizing access to ROS targets in an aqueous environment. In contrast, surface functionalization that imparts a positive zeta potential can amplify pro-oxidant activity by facilitating electrostatic attraction to the anionic membranes of cells and mitochondria. This targeted localization triggers membrane disruption and facilitates overwhelming cellular uptake, culminating in severe, localized oxidative stress. Overall, ML offers a distinct advantage over traditional methods by elucidating complex, nonlinear relationships and synergistic effects that are often obscured in conventional analysis. Unlike simpler models, ML algorithms can capture intricate patterns such as sharp activation thresholds, optimal “sweet spots,” and exponential dependencies. This capability allows for the development of models that more faithfully represent the underlying physics and chemistry, leading to significantly greater predictive accuracy.

The studies discussed above demonstrate that ML techniques can minimize NM toxicity by offering mechanistic insights into their adverse effects. This knowledge supports the development of effective hazard and risk assessment strategies, promoting the safe-by-design principle for NMs. Nevertheless, the implementation of ML approaches also presents several challenges that require careful consideration. The following sections outline these challenges and discuss strategies to address them.

### 5.1. Data Deficiency and Lack of Standardized, Curated Datasets

One of the foremost challenges in applying ML to nanotoxicology lies in data scarcity. Existing datasets often contain missing or incomplete values, whereas robust ML models require large, high-quality datasets for reliable prediction.

To address this limitation, read-across approaches have been widely adopted, enabling the inference of toxicity for untested NMs based on available data from structurally or functionally similar substances. For instance, multi-nano-read-across modeling has been employed in nano-safety assessment, where hybrid strategies combining quantitative analysis with ML methods facilitated the simultaneous evaluation and prediction of the toxicity of metal and silica oxide NPs [68]. By classifying NPs according to shared structural and physicochemical characteristics, this method facilitates the early identification of hazardous materials and supports the safe design of NMs.

Grouping has thus emerged as a promising strategy to tackle data gaps without requiring extensive new experimental studies, which is a process of classifying substances into a common group based on their structural similarities and shared or predictable physicochemical, toxicological, and environmental interaction properties, thereby enabling the prediction of effects for unknown substance using data from related materials [86]. In particular, omics-based grouping combined with predictive ML models has streamlined the laborious conventional hazard evaluation by leveraging similarities among NMs [87,88,89].

Another effective solution to data scarcity is data augmentation, which not only expands limited datasets through the generation of synthetic data, but also mitigates model overfitting [90]. Recent work has applied deep learning methods, such as the Mask R-CNN algorithm, to segment images of vanadium pentoxide (V_2_O_5_) nanowires obtained via diverse microscopy techniques [91]. To compensate for limited experimental data, synthetic image datasets replicating the geometric and visual characteristics of real nanostructures were generated, thereby improving model training. This strategy is not limited to V_2_O_5_ but is adaptable to a wide range of materials, offering broad utility in materials science and nanotoxicology.

Furthermore, the field of nanotoxicology is currently hindered by the lack of large-scale, high-quality, and standardized public datasets. Data is scattered across thousands of publications, showing significant heterogeneity in nanomaterial characterization, dose metrics, and endpoint measurements. The absence of standardized protocols for measuring physicochemical properties or conducting toxicity assays often results in substantial variability and conflicting outcomes, thereby complicating comparisons across different studies. This data fragmentation and the inherent biases, such as selection and measurement bias, in training datasets, severely challenge robust model training and, crucially, generalizability of ML Models. Consequently, there is an urgent need for a community-wide initiative to establish and maintain high-quality, and standardized open-access databases for nanotoxicology. These databases should include detailed metadata on NM physicochemical properties, comprehensive experimental protocols, and raw data from various assays. To address this issue, quality criteria for NM experimental data have been proposed to enhance reliability and enable integration across diverse datasets [92,93]. In parallel, microfluidics-based synthesis has emerged as a powerful tool to improve data quality and reproducibility. By enabling precise control over parameters such as flow rates, mixing rates, and reaction volumes at the micro/nanoscale, microfluidic platforms provide highly reproducible NM synthesis and can be seamlessly integrated with characterization systems [94]. Such standardization not only enhances data quality but also supports the generation of consistent datasets for ML-driven toxicity prediction. Such datasets are critical for democratizing ML development. They would establish a gold standard for benchmarking new models and, most importantly, serve as the foundation for creating truly generalizable predictive tools that are urgently needed in the field.

### 5.2. Algorithm Enhancement

Alongside data-related challenges, the evolution of computational methods represents both an opportunity and a necessity for advancing predictive nanotoxicology. Traditional ML approaches, while effective in pattern recognition and regression, may not fully capture the nonlinear and high-dimensional nature of NM–biological interactions. With the rapid growth of data availability and improvements in computational resources, deep learning architectures such as CNNs and recurrent neural networks (RNNs) are increasingly being explored. These models can automatically extract hierarchical features from raw data, making them particularly suitable for tasks such as NMs risk assessment, high-throughput data curation, and image-based analysis [95]. For example, CNNs have been successfully applied to transmission electron microscopy and scanning electron microscope images to automatically detect NMs and quantify their size and abundance [96], with potential extension to dynamic video analysis. Moreover, combing different neural network architectures or integrating them with other ML methods can leverage complementary strengths, thereby enhancing predictive accuracy and interpretability.

It is also important to note that most current ML frameworks primarily depend on static, endpoint toxicity data, such as ROS levels measured at 24 h. However, ROS generation is inherently dynamic, and understanding its kinetics may provide more insight than a single endpoint measurement. Additionally, integrating complex, time-resolved data from in vivo studies—which includes factors like pharmacokinetics, biodistribution, and systemic immune responses—into predictive models remains a formidable challenge. Existing models generally fail to capture these temporal dynamics, offering only a simplified view of complex biological interactions. To accurately capture the dynamics of ROS generation, future research should aim to integrate ML with high-throughput, real-time experimental platforms. Technologies such as microfluidics, exemplified by organ-on-a-chip systems, coupled with high-content imaging and fluorescent biosensors, can provide rich, time-series data on cellular responses to nanomaterial exposure. ML models, especially those designed for sequential data like Recurrent Neural Networks (RNNs), could then be trained to predict toxicity kinetics and decipher dynamic structure-activity relationships. Such advances are expected to accelerate automated, high-throughput exploration of NM-ROS interactions and facilitate the design of safter NMs.

In addition, the reviewed articles frequently employed SHAP (Shapley Additive Explanations) for model interpretation. As a game-theoretic method, SHAP computes the precise contribution of each feature to an individual prediction. This preference is often attributed to several key advantages: its guarantee of local and global consistency, and the flexibility of its model-agnostic KernelSHAP implementation. Despite these strengths, SHAP has a critical theoretical limitation: the assumption of feature independence. This is particularly relevant for the model-agnostic KernelSHAP and PermutationSHAP methods, which implicitly assume a feature’s value can be changed in isolation without affecting others. In real-world scenarios where features are often correlated (e.g., a nanoparticle’s primary size and its surface area), this assumption is violated. Consequently, SHAP may produce unreliable explanations for these features, often by incorrectly attributing importance to one feature over another or by splitting the contribution between them, rendering the explanations untrustworthy. To mitigate this issue, it is essential to analyze feature correlations prior to interpretation and to exercise caution when evaluating the importance of co-dependent features. When its limitations are properly considered, SHAP analysis transitions from a simple explanatory tool to a powerful engine for hypothesis generation. For instance, if the analysis revealed a distinct threshold effect for zeta potential, where it becomes a strong predictor of toxicity only above approximately +15 mV. This finding led to the mechanistic hypothesis that a sufficiently high positive surface charge is required to overcome electrostatic repulsion with the cell membrane, leading to strong binding and subsequent membrane disruption. This hypothesis can be tested experimentally by synthesizing NPs with identical cores and sizes but with zeta potentials tuned across this +15 mV threshold. The hypothesis would be validated if particles above +15 mV induce a sharp drop in cell viability (WST-1 assay) that directly correlates with increased membrane damage (LDH release assay).

The ultimate vision is to leverage ML not just for prediction, but also for de novo design. This involves creating “inverse design” or “generative” models that, given a desired toxicity profile (e.g., minimal ROS for a drug delivery vehicle, or maximal ROS for a cancer therapeutic), can propose novel NM structures with the target properties. By coupling these generative models with active learning, where the model intelligently suggests the most informative experiments to perform next, researchers can create highly efficient, closed-loop “self-driving labs” for accelerating the design of safer and more effective NMs.

### 5.3. Translational Barriers to Real-World Application

Beyond intrinsic technical gaps, formidable translational barriers impede the adoption of these models in clinical and regulatory settings. Foremost among these is the lack of standardized frameworks to empirically validate ML predictions. In silico hypotheses must be rigorously tested in clinically representative systems (e.g., patient-derived organoids, preclinical animal models) and environmentally relevant contexts. This empirical validation loop is indispensable for demonstrating the real-world reliability of ML-driven assessments. In its absence, ML models risk remaining isolated within academic discourse, failing to mature into practical tools for safety and risk assessment.

The path to regulatory endorsement is equally challenging, primarily due to the issue of model opacity. Regulatory bodies such as the FDA, EPA, and ECHA operate on a foundation of transparent, reproducible, and validated test guidelines (e.g., OECD protocols). The inherent “black box” nature of many sophisticated ML algorithms, especially deep neural networks, is fundamentally at odds with this regulatory paradigm. Therefore, the adoption of Explainable AI (XAI) is essential. XAI methodologies can deconstruct complex models to provide mechanistic insights into their predictions, thereby fostering the trust required for their integration into regulatory frameworks as qualified New Approach Methodologies (NAMs) that advance the 3Rs principle (Replacement, Reduction, and Refinement of animal testing).

## 6. Conclusions

ML algorithms are highly effective in recognizing complex patterns and generating reliable predictions from large datasets, making them particularly valuable for elucidating and tailoring the intricate properties of NMs. The integration of ML into nanomaterial research has the potential to accelerate discovery and optimization, thereby enabling more efficient design and synthesis. Although significant challenges persist, such as data scarcity, heterogeneity, and model interpretability, the transformative potential of ML-driven strategies to overcome these limitations highlights their critical role in shaping the next generation of nanotechnology. Ultimately, this convergence of ML and nanoscience is expected to unlock new applications and enhance existing technologies, paving the way for transformative advances in predictive nanotoxicology and safe-by-design NMs that extend beyond current technological boundaries.

## Figures and Tables

**Table 1:** The summary of various parameters mentioned in the reviewed literature related to the induction of ROS by NMs.

ENMs	Endpoints	In Vitro/ In Vivo	Algorithms	Data Size	Descriptors	Performance	Ref.
MeOx	DCFH-DA assay	*C. elegans*	MC-PLS	16 MeOx NMs	quasi-SMILES descriptors (mass percentage of metal elements, cationic charge and MW, Initial size, aggregation size, fP)	N/A	[48]
nTiO_2_	Oxidative stress biomarkers (CAT, GPx, GST, MDA, ROS, and SOD)	bivalves	RF, ANN, KNN, XGB, SVC, Gaussian NB	32 relevant references	physicochemical descriptors and experimental variables (NP size, exposure conc., intervals of time exposed to NPs, dissimilar assay organisms/tissues/organs)	accuracy varied from 0.75 to 1.00 (training sets) and 0.60 to 1.00 (external validation sets)	[49]
Nano-TiO_2_-heavy metals (CdCl_2_, ZnCl_2_, MnCl_2_, CoCl_2_, CuSO_4_, NiCl_2_, Pb(NO_3_)_2_, SbCl_3_)	CCK-8 assay	HK-2 human renal cells	PLS, RF, AdaBoost, K-means clustering	72 total samples (8 heavy metals and 9 concentrations mixed with nanoTiO_2_)	orbital energies, ionization potential, electron affinity, electronegativity, hardness, molecular and adsorption energy	0.31 < R^2^ < 0.95 with clustered RF performing best	[50]
ZnO	Luciferase assays	cells	sparse MLR (MLREM), Bayesian regularized artificial neural networks (BRANNLP)	45 types of ZnO NPs	conduction band energy, reduction potential, ionization potential, fP, solubility, percentage of metal oxide dopant, surface coating type, calcination temperature, NP size, surface area, volume, and aspect ratio.	0.50 < R^2^ < 0.67	[51]
Superparamagnetic iron oxide NPs	N/A	N/A	PLSR	74 types of SPIONs	experimentally obtained surface properties (size, shape, surface charge, coating type and functional groups) with molecular descriptors MW, TPSA Wiener Index (W) LogP 3D Autocorrelation)	(R^2^ = 0.9515) and MSE of prediction (MSECV = 0.6270)	[52]
MeONPs (e.g., Cr_2_O_3_, CoO, MnO_2_, CuO, ZnO, Ni_2_O_3_, Co_3_O_4_, etc.)	MTS assay	THP-1 cells	PR, MPR, GMPR, SVM, RF, kNN, GkNN, DT, BR, NN + RF	240 observations (30 types of MeONPs and 8 concentrations)	size, charge, ion release, electronegativity, concentration	R^2^ values ranged from as low as –1.00 (SVM, poor generalization) to as high as 0.90 (GkNN, excellent prediction)	[39]
Gold NPs	H_2_O_2_ level	cells	RF, kNN	191 unique GNPs	geometrical nanodescriptors	moderate accuracy: R^2^ = 0.68	[56]
Gold NPs	HO-1 level	cells	kNN	34 GNPs	chemical descriptors	R^2^ = 0.967 and MAE= 0.14	[57]
Silver NPs	MTT/MTS, LDH, SRB, NRU, and trypan blue	cells	DT, RF	690 data points	particle size, fP, exposure dose, exposure time, cell type, species, plant family, extraction solvent	accuracy improved from ~0.73 (DT1) up to 0.83 (RF2), with AUC ranging 0.815–0.904, especially for RF with biosynthesis features included	[58]
MXenes	MTT assay/CCK-8 assay/DCFH-DA	cells	Regularized Logistic Regression, RF, SVM, ERT	132 records	surface chemistry, modifications, morphology, and synthesis conditions, with MxOy and Li presence as the strongest predictors of cytotoxicity.	SVM-rbf, RF achieved ~0.85–0.93 accuracy, while purely theoretical features performed poorly.	[59]
CDs	H_2_O_2_ content	Hairy root culture	MLP	N/A	metabolite	MSE = 1.99 × 10^−3^, R^2^ = 0.99939	[64]
Graphene	ROS generation	cells	PLSR	11 2DNMs	geometrical nanodescriptors	R^2^ = 0.760, RMSE = 0.164	[65]
Fullerene, Graphene Oxide, SWCNTs, MWCNTs, Graphene Nanosheets	DCFH-DA assay	cells	Classification: C4.5 DT, SVM, ANN, NB, kNN; Regression: DT, RF, GB, Adaboost.	N/A	physical and chemical descriptors: fP, DH, and SSA	classification models: performance metrics (Accuracy, Recall, Precision, F1-score) all exceeding 0.600; regression models: 0.850 < R^2^ < 0.999 on the training set	[66]
Organic and inorganic	Ferric reduction ability of serum assay	in human blood serum	DT	19 NMs	285 structural descriptors (both experimental and calculated)	100% of balanced accuracy	[67]
Inorganic (metal- and silica oxide)	Viability test	cells, bacteria, algae, and protozoa.		184 inorganic (30 unique types) NPs	ionic characteristics		[68]
Inorganic NMs (TiO_2_, ZnO, silver and silica)	The level of oxidized base lesions (oxidatively damaged DNA)	cells	the supervised PLS method	17 JRC repository NMs	physicochemical descriptors	N/A	[69]
Inorganic NMs (metals and metal oxides)	Cell viability, concentration-dependent toxicity	cells	LightGBM regressor, RF, ET, HGB, Binary Relevance	3087 samples	atom-based, nanoparticle physicochemical, and experimental condition descriptors	LGBM show s the best overall; Q^2^ = 0.86, RMSE = 12.2%	[70]
Metallic (Au, Ag), metal-oxide (ZnO, TiO_2_, CuO, CoFe_2_O_4_, Fe_2_O_3_), polymeric (polystyrene), and SiO_2_ NPs	N/A	cells	DT, RF, SVM, NB, ANN	244 records (NanoHub repository)	physicochemical properties, exposure conditions, cell model characteristics	Random Forest showed superior performance (AUC ≈ 0.97, accuracy > 93%), outperforming DT, SVM, NB, and ANN.	[71]
Metal and metal oxide NPs (e.g., Ag, TiO_2_, ZnO, Au, Pt, CuO)	N/A	cells	41 regression ML models	3627 samples with 27 features	NPs concentration, fP, hydrodynamic diameter, exposure time, electronegativity of central atom, cell type/line identify	tree-based ensemble models (LGBM, XGB) clearly outperformed all others, with LGBM optimized to the best accuracy (Q^2^~0.80).	[72]

**Table 2:** Summary of the various parameters mentioned in the reviewed literature related to the scavenging of ROS by NMs.

ENMs	Endpoints	In Vitro/ In Vivo	Algorithms	Data Size	Descriptors	Performance	Ref.
(La, Sm)-doped ZnO NPs	DCFH_2_-DA assay	cells	LR, MLP, RF, DTs, ETs, KNNs, GB, SVR	196 observations	material, grain size, TC, BG, defects, charge, average particle size, method, and Conc.	LR: RMSE = 22.28; RF: RMSE = 13.43; ET: RMSE = 16.12; DT: RMSE = 16.07; MLP: RMSE = 31.4; KNN: RMSE = 33.09; GB: RMSE = 13.19; SVR: RMSE = 32.93.	[73]
(Er, Yb)-doped ZnO NPs	DPPH scavenging (%), ABTS scavenging (%), H_2_O_2_ scavenging (%).	in vitro	LR, RF, ET, DT, MLP, KNN, GB, SVR	480 observations	NP Conc., optical BG, SSA, fP, Zn, Er, Yb composition	ET: R^2^ = 98.92 DT: R^2^ = 98.21 RF: R^2^ = 96.89 GB: R^2^ = 94.76 MLP: R^2^ = 91.88 KNN: R^2^ = 84.93 LR: R^2^ = 78.54 SVR: R^2^ = 70.83	[74]
Nd-doped CeO_2_ NPs	DPPH and ABTS assays	cells	RF, GB, logistic regression, MLP	144 observations	molarity, MW, lattice constant, fP, SSA, Ce^3+^/Ce^4+^ ratio, and scavenging activity.	RF: accuracy = 96.35%, GB: accuracy = 96.47%, LR: accuracy = 92.67%, MLP: accuracy = 88.33%	[75]
Different inorganic and organic NMs	DPPH assay	In vitro	regression models (RF, ET, LIGHTGBM, DT, KNN, LASSO, EN).	62 in vitro studies	P-chem properties, exposure conditions and the method of NMs’ synthesis (NMs’ type, core size, shape, dosage, coating, the synthesis process, medium used, absorbance and duration)	RF: R^2^ = 0.83. ET: R^2^ = 0.79 LIGHTGBM: R^2^ = 0.81 DT: R^2^ = 0.76 KNN: R^2^ = 0.70 LASSO: R^2^ = 0.52 EN: R^2^ = 0.30	[76]
2D Nanozymes	Peroxidase- and Catalase-like activity	N/A	XGBR, LR, Ridge regression, SVM, KNN, GBR, RF, NEM	1019 2D materials	atomic number, valence electrons, electronegativity, ionization energy, atomic radius, electron affinity	XGBR achieved the highest accuracy (R^2^ ≈ 0.85–0.97)	[80]
Bimetallic NPs	Catalytic dissociation of H_2_O_2_	N/A	CatBoost regression	1260 bimetallic alloy structures	structure descriptor	R^2^ = 0.964, MAE = 0.108, RMSE =0.169.	[81]
Nanozymes	CAT-like activity	N/A	RF, XGBoost, AdaBoost, MLP, CNN	over 100 types of NMs	shape, size, metal type, the number of valence electrons for each metal, nonmetallic elements, and surface modification techniques.	RF: accuracy = 62% XGB: Accuracy = 58% AdaBoost: Accuracy = 67% MLP: Accuracy = 78% CNN: Accuracy = 80%	[82]
Inorganic NMs	H_2_O_2_ activation, H_2_O_2_ dismutation, O_2_ activation, and O_2_^·−^ dismutation	N/A	XGBoost regression	1019 materials	adsorption energies, electronic structure, reaction energy and energy barriers	N/A	[83]

## Data Availability

Not applicable.

## Abbreviations

The following abbreviations are used in this manuscript:

R^2^	coefficient of determination
MSE	mean squared error
RMSE	root mean square error
MAE	mean absolute error
MC-PLS	Monte Carlo partial least squares
PLSR	partial least squares regression
PLS	partial least squares
RF	random forest
DT	decision tree
GB	gradient boosting
SVM	support vector machine
NB	naive bayes
ETs	extremely random trees
EN	elastic net
MLP	multilayer perceptron
ANN	artificial neural network
LR	linear regression
KNN	k-nearest neighbor
SVR	support vector regressor
XGB	extreme gradient boosting
LightGBM	light gradient boosting machine
AdaBoost	adaptive boosting
CNN	convolutional neural networks
NEM	neural network models
TC	texture coefficient
BG	bandgap
SSA	specific surface area
fP	zeta potential
MW	molecular weight
TPSA	topological polar surface area

## Appendix A

**Table A1:** Statistical summary for the machine learning algorithms used across the studies.

RF	GB	ANN	DT	KNN	SVM	PLS	ETs	LR	NB	Adaboost	Logistic Regression	Others
16	14	11	9	8	8	5	5	4	3	3	2	7

**Table A2:** Statistical summary for the extrinsic factors influencing oxidative stress mentioned in the reviewed articles.

Dosage	Exposure Regime	Cell/Tissue Type	Experiment Method	Plant Extracts for Synthesis
9	4	3	2	1
